# Metastatic melanoma of the major salivary glands - a systematic review

**DOI:** 10.4317/medoral.27216

**Published:** 2025-08-16

**Authors:** Pedro Henrique Rizzardi, Amanda de Farias Gabriel, Isabela Domingues Martins, Vinicius Coelho Carrard, Virgílio Gonzalez Zanella, Fábio Muradás Girardi, Marco Antonio Trevizani Martins, Vivian Petersen Wagner, Manoela Domingues Martins, Lauren Frenzel Schuch

**Affiliations:** 1 DDS, Department of Oral Pathology, School of Dentistry, Universidade Federal do Rio Grande do Sul, Porto Alegre, Rio Grande do Sul, Brazil; 2 DDS, Department of Oral Pathology, School of Dentistry, Universidade Federal do Rio Grande do Sul, Porto Alegre, Rio Grande do Sul, Brazil; 3 Nursing Student, Michigan State University, East Lansing, Michigan, United States; 4 DDS, PhD, Department of Oral Pathology, School of Dentistry, Universidade Federal do Rio Grande do Sul; Department of Oral Medicine, Hospital de Clínicas de Porto Alegre, Universidade Federal do Rio Grande do Sul, Porto Alegre, Rio Grande do Sul, Brazil; 5 MDM, Department of Head and Neck Surgery, Hospital Santa Rita, Santa Casa de Misericórdia de Porto Alegre, Porto Alegre, Brazil; 6 MDM, Department of Oral Pathology, School of Dentistry, Universidade Federal do Rio Grande do Sul, Porto Alegre, Rio Grande do Sul, Brazil; 7 DDS, PhD, Department of Oral Pathology, School of Dentistry, Universidade Federal do Rio Grande do Sul; Department of Oral Medicine, Hospital de Clínicas de Porto Alegre, Universidade Federal do Rio Grande do Sul, Porto Alegre, Rio Grande do Sul, Brazil; 8 DDS, PhD, Department of Dentistry, School of Dentistry, Universidade de São Paulo (FOUSP), São Paulo, Brazil; 9 DDS, PhD, Department of Oral Pathology, School of Dentistry, Universidade Federal do Rio Grande do Sul; 10 DDS, PhD, Department of Diagnosis in Pathology and Oral Medicine, Universidad de la Republica (UDELAR), Montevideo, Uruguay

## Abstract

**Background:**

Metastatic melanoma of the major salivary gland has been rarely reported in the literature. Thus, the aim of this study was to integrate all data about the clinical, sociodemographic, histopathological, treatment, and follow-up characteristics of metastatic melanoma of the major salivary glands.

**Material and Methods:**

Electronic searches were performed in five databases and the grey literature according to the Preferred Reporting Items for Systematic Reviews and Meta-Analyses (PRISMA) 2020. Case reports or case series describing metastatic melanoma of the major salivary gland without language or year of publication restriction were included.

**Results:**

Twenty-five studies reporting 47 cases of metastatic melanoma were identified. The mean patient age at diagnosis was 56.321.5 years, mainly occurring in men (70.2%). The site of the primary melanoma was mostly in the head and neck region (65%) and the parotid (93.6%) was the most affected major salivary gland by metastatic lesions. Parotidectomy was the main treatment choice. The overall 1- and 5-year survival rates were 79% and 45%, respectively.

**Conclusions:**

Metastatic melanoma of the major salivary gland is an uncommon phenomenon involving a poor prognosis.

** Key words:**Melanoma, head and neck neoplasm, metastasis, parotid, major salivary glands.

## Introduction

Melanoma, a malignancy originating from melanocytes, manifests a variety of clinico-pathological subtypes in both sun-exposed and non-sun-exposed regions. In 2020, there were 324,635 new cases worldwide, resulting in 57,043 deaths (1). Morbidity and mortality are primarily linked to metastatic disease, even from small primary tumors (2). While many patients are cured following excision of the primary tumor, recurrence, often occurring at distant sites, is associated with a poorer prognosis (3).

According to the literature, approximately 15 to 25% of all primary cutaneous melanomas occur in the head and neck region, exhibiting a less favorable prognosis compared to melanomas originating from other anatomical sites (3-5). The major salivary glands and their associated lymph nodes can occasionally be affected by metastases from head and neck carcinomas as well as cutaneous melanomas (6). Treatment for metastatic major salivary glands from cutaneous melanoma often involves surgery, neck dissection, and adjuvant therapy. In recent years, advancements in treatment options, such as immunotherapy, have notably enhanced the prognosis and outcomes for individuals with melanoma (7).

Given the necessity for comprehensive data regarding the metastatic melanoma involving the major salivary glands, this systematic review was undertaken to address the following inquiry: What are the clinicodemographic characteristics, overall survival, and prognostic factors associated with metastatic melanoma in the major salivary glands?

## Material and Methods

- Eligibility criteria

The inclusion criteria for the present systematic review established using the PECOS method, as follows: (P) individuals diagnosed with metastatic melanoma in a major salivary gland (parotid, submandibular or sublingual); (E) metastatic melanoma; (C) not applicable; (O) clinicopathological data pertaining to metastatic melanoma in the major salivary glands; and (S) case reports and case series. No language restriction was applied. A true metastasis was defined based on specific criteria: the metastatic lesion should be distinguishable from direct invasion by the primary cancer, histologically identical to the primary lesion, and recognized as originating from the primary site of the tumor (8).

Excluded reasons were: 1) literature reviews, *in vitro* or animal studies; 2) reports of primary melanomas in a major salivary gland; 3) melanomas of cervical lymph nodes; 4) cases that do not describe the primary site; 5) longitudinal studies where specific information about individual cases could not be collected.

- Sources of information and search strategy

Systematic searches with no publication date restriction were undertaken in February 2024 in the following electronic databases: PubMed, Scopus, Web of Science, Embase, and LILACS. An additional search was conducted in the grey literature (Google Scholar and ProQuest). The search strategy was formulated utilizing a combination of MeSH terms, entry terms, and free terms, with detailed information provided in Supplement 1. Manual searches were also conducted by reviewing the reference lists of the included articles to identify any publications that may have been overlooked during the electronic database searches. EndNote X9 (Thomson Reuters, Philadelphia, PA) was used to manage all the references recorded in the selection process of this study.

- Study selection

The studies underwent a two-phase selection process. Initially, two reviewers (P.H.R. and L.F.S.) screened the titles and abstracts of the articles. Studies that did not meet the inclusion criteria were automatically excluded. The reviewers were calibrated based on their evaluation of the first 50 retrieved references, resulting in a kappa value of 0.94. In the second phase, the same reviewers evaluated the full text of the studies selected in the first phase. Articles that m*et al*l inclusion criteria were ultimately included in this systematic review. Any discrepancies between the two authors during these phases were resolved by seeking consultation from a third (M.D.M.), more experienced reviewer.

- Data collection process

The following data were extracted on a standard form by two independent reviewers (P.H.R. and L.F.S.): 1) information regarding author, country and publication data; 2) patient’s age and sex; 3) site of primary melanoma (and time between primary and metastatic lesion); 4) anatomical location of the metastatic lesion; 5) clinical presentation; 6) reported symptoms and duration; 7) size (cm); 8) imaginological findings; 9) management; 10) histopathological and immunohistochemical findings; 11) treatment; 12) recurrence; 13) follow-up period; and 14) status (dead or alive).

- Critical appraisal

Critical appraisal of the included articles was carried out using the Joanna Briggs Institute - University of Adelaide tool for case reports or case series (9). This tool encompasses the evaluation of various aspects of reporting quality to assess bias risk, such as patient history, demographic data, diagnosis, and intervention. Each question elicited a response indicating whether the study met the specified criteria (yes), did not meet them (no), if there was uncertainty (unclear), or if the criteria did not apply to that study (not applicable).

- Synthesis methods

The extracted data underwent initial descriptive analysis using Microsoft Office Excel 2019 (Microsoft® software, Redmond, WA, USA). Subsequently, survival data and status were analyzed with Prism 8 software for Mac, version 8.4.3 (GraphPad Software, Boston, Massachusetts, USA, www.graphpad.com). Kaplan-Meier survival curves were generated from the collected data and compared using the Log-rank (Mantel-Cox) test, with statistical significance set at *p* < 0.05. To ensure an adequate sample size for analysis, continuous variables such as age and time between primary diagnosis and metastasis were dichotomized. Additionally, the site of the primary tumor was recategorized into two main categories: head and others.

## Results

- Study selection

The electronic search yielded 3,181 articles; 59 full-text articles were evaluated. Forty studies were excluded (Supplement 2), and 19 articles were selected. Additionally, six studies were included from grey literature and references cross-checking (Supplement 3). Ultimately, 25 articles reporting 47 cases were included (Supplement 4).

- General characteristics of the included studies

The studies included in the analysis spanned the years 1977 to 2022 and were conducted in 12 different countries. Most reports originated from the United States of America (7 articles), followed by the United Kingdom (4 articles), and Turkey (3 articles). Detailed extraction data are provided in Supplement 5. Table 1 presents summarized information on the 47 cases.

- Demographic data

Males accounted for the majority (*n*=33/70.2%) of cases, with patient ages ranging from 5 to 85 years (mean: 56.3 ± 21.5). Cases were often observed between the sixth and eighth decades of life (*n*=31).

- Site of primary melanoma, anatomical location of the metastasis, clinical features, symptoms, and time course

**Figure 1:**
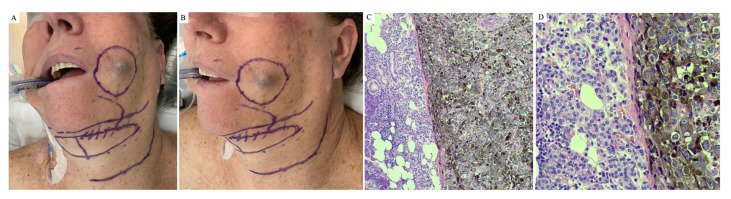
An illustrate clinical case of a left cheek melanoma (confirmed by histopathological analysis and positive staining for SOX10, MelanA and HMB-45) in a 60-female patient, with metastasis on left parotid. A, clinical appearance of a black nodule on the left side of face skin, measuring 2.2x1.6cm; B, notable swelling on the left parotid, firm on palpation, without skin erythema; C and D, hematoxylin and eosin (H&E) stained images with notable melanoma characteristics reveal densely clustered melanoma cells exhibiting atypical nuclei and melanin pigmentation.

Forty primary melanomas occurred on the head and neck region. Fig. 1 illustrates a case of head and neck cutaneous melanoma with parotid gland metastasis. Among 31 cases with available information, metastatic lesions preceded the melanoma diagnosis in six cases (19.4%). In the remaining 25 cases, the mean time between the primary tumor and metastasis was 2.5 ± 2.1 years (ranging from 1 month to 9.3 years).

The parotid gland was affected in nearly all cases (93.6%), with only three cases occurring in the submandibular gland. No metastasis to the sublingual gland was identified. The clinical presentation commonly involved swelling, ranging from 0.18 to 6 cm (mean: 2.5 ± 1.3 cm). Among the cases with reported symptoms (*n*=14), 42.9% were painful, with symptom duration ranging from 0.5 to 12 months (mean: 4.6 ± 3.8 months). The mean age for patients without and with pain at presentation was 58.5 and 58.6 respectively, with no significant difference between groups (*p*=0.85, Mann-Whitney test). Information about pain and time elapsed between primary tumor and the metastatic lesion was present in 11 cases. The mean time elapsed for patients without symptoms was 1.68 (standard deviation 2.34) and for those with pain was 2.24 (standard deviation 2.18). The difference was not statistically significant (*p*=0.66, Mann-Whitney test).

- Auxiliary diagnostic methods, imaginological exams, and histopathological and immunohistochemical findings

 Fine needle aspiration cytology (FNAC) was performed in 22 of 25 informed cases as an auxiliary diagnostic method. Imaging exams were performed in 14 cases: sialography, computed tomography (CT), magnetic resonance imaging (MRI), positron emission tomography (PET) scan, and ultrasonography.

Histopathological and immunohistochemistry aspects were detailed in 13 and 17 articles, respectively (Table 2). In general, descriptions included features such as fasciculate cells with large pleomorphic nuclei, prominent nucleoli, neoplastic cells, mitotic Figures, eosinophilic cells, and multinucleated giant cells. Some cases also exhibit brownish and melanin-rich pigments. There was no significant difference between cell morphology and the survival time (*p*=0.71, log-rank test). Table 3 summarizes the immunostaining results of the informed cases.

- Treatment, follow-up, and patient's status

**Figure 2:**
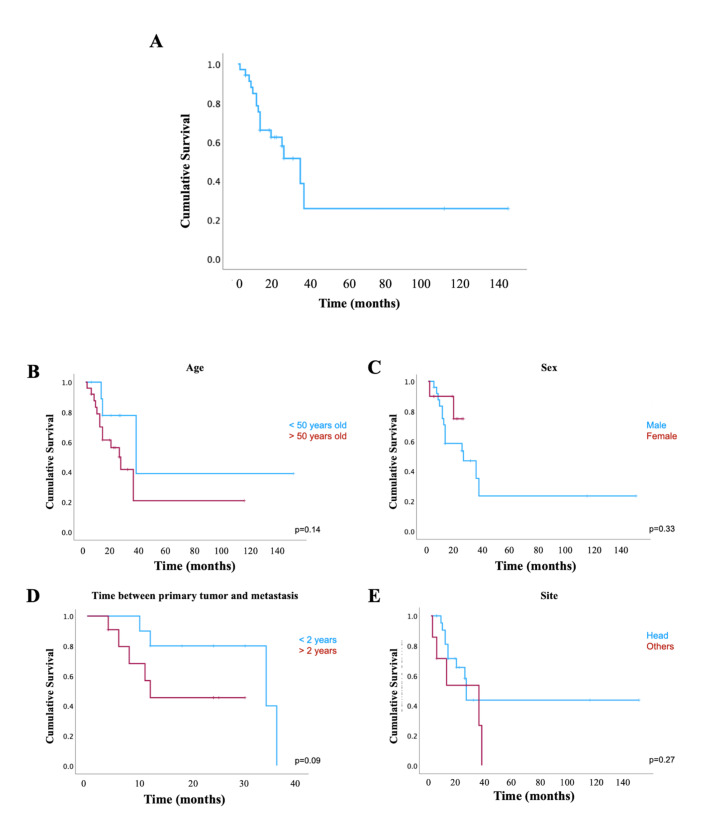
Statistical analysis. Kaplan Meier overall survival curve (A). Survival curves according to (B) gender, (C) age, (D) time between primary tumor and metastasis, (E) site of primary tumor.

Total/radical parotidectomy was the most commonly utilized treatment, often combined with neck dissection. Recurrence was observed in 2 out of 14 informed cases, occurring at 7- and 9-months post-treatment. Follow-up was provided in 36 reports, with an average monitoring duration of 22.8 ± 28.4 months, ranging from 1 to 148 months. Sixteen patients (41%) died. Thirty-five cases reported information on both survival status and time, which allowed survival analysis. Only three patients had a follow-up longer than 3 years. The overall 1-,3- and 5-year survival rates were 79%, 52% and 45%, respectively (Fig. 2).

Four features were investigated regarding its prognostic value (due to data availability) (Fig. 2). Sex, age (cut off 50 years old), site of primary tumor (head vs other sites, including neck), and the time between primary diagnosis and metastasis were not significantly associated with survival.

- Critical appraisal

The results of all the questions applied in the critical appraisal are presented in Supplement 6.

## Discussion

Metastatic melanoma of a major salivary gland is an uncommon condition and, to the best of our knowledge, only forty-seven cases published in the literature between 1977 and 2022 were found. Saravakos *et al*. (10) conducted a retrospective study with 1,020 cases of parotid lesions. Among 79 cases of metastatic tumors, only four were melanoma, representing 0.4% of the total sample. Despite its rarity, Bhaat *et al*. (11) reported that melanoma was the second most common solid tumor to metastasize to the parotid gland, emphasizing the need for further investigation into clinicopathologic scenarios and prognostic markers. These data underscore the importance of the present systematic review.

Most cases of metastatic melanoma in major salivary glands occurred in males aged between the sixth and eighth decades of life. Due to the rarity of these lesions, demographic data in the literature are limited. Bron and coauthors (12) found a predominance of men and a mean patient age of 65 years in their study on primary and metastatic cancers of the parotid gland. The head and neck region is commonly reported as the primary site of metastatic melanoma (13), consistent with our findings where almost all cases occurred in this region. The parotid gland, responsible for nearly all metastatic cases in the included studies, serves as a lymphatic drainage filtering station for the head and neck region, potentially explaining the high number of cases originating from this area (10,14).

The predominant clinical presentation was swelling (100%), often self-detected by patients prompting medical attention. However, symptom reporting was inadequate, with 8 patients experiencing painless conditions and 6 cases reporting pain. According to Guzzo *et al*. (15), malignant salivary tumors exhibit a spectrum of biological behaviors, with approximately 40% of these tumors displaying indolent characteristics, particularly among young individuals. These tumors typically manifest as slow-growing lumps and, if present for an extended period, may be associated with pain or early nerve involvement. The interval between the detection or treatment of the primary lesion and the onset of symptoms related to the salivary gland region varied widely, ranging from 1 month to 9.3 years. Furthermore, when associating the time elapsed between the initial diagnosis and metastasis with pain, no significant difference was observed, likely due to the small number of cases.

Regarding diagnostic methods, FNAC was used in 22 cases included in our review. Studies have described FNAC as a noninvasive, low cost and painless method, with few complications, representing an accurate test for the diagnosis of melanoma metastases. The analysis is usually fast, thus facilitating the diagnosis of these lesions (16-18). Images were used to aid diagnosis and described in 14 cases. It is important to highlight that patients with palpable masses that have been clinically identified require an imaging exam. In this review, MRI was the exam most frequently used along with CT. In this review, MRI was the exam most frequently used along with CT. While MRI and CT are recognized in the literature as precise, sensitive, and specific diagnostic tools, a meta-analysis revealed that ultrasound exhibited greater sensitivity and specificity in staging and follow-up among patients with melanoma (19). Particularly noTable is ultrasound is significant enhancement in absolute sensitivity compared to clinical examination alone for diagnosing locoregional melanoma metastasis, as highlighted in a recent meta-analysis (20). In contrast, PET-CT demonstrated superior sensitivity and specificity in detecting distant metastases (21). It is noteworthy that PET-CT was utilized in only two cases, whereas ultrasound was employed in five within this review.

From a histological perspective, melanoma shows proliferation of neoplastic melanocytes with different cell types, including epithelioid cells and fusiform and plasmacytoid tumor cells (22). In the present review, several cellular phenotypes were described in metastatic melanoma lesions, among them fasciculate cells with large pleomorphic nuclei, prominent nucleoli, neoplastic cells, mitotic Figures, eosinophilic cells, and multinucleated giant cells. The presence of melanin in the cells is important by contributing to the histological diagnosis (23); however, few reports described some type of melanic pigment in the cells. There is also the existence of amelanotic melanoma, which has little or no pigmentation and whose histological characteristics are mainly a high mitotic rate, epithelioid cells, spindle cells, small cells, rhabdoid cells and solar elastosis as a strong predictor of amelanotic melanoma (24). Immunohistochemical markers are extremely important for the definitive diagnosis of these lesions. Positive markers for S-100, vimentin, HMB-45, Melan-A/MART-1 and negative markers for cytokeratin are likely to be reported in the literature (22,23). In this review, 17 studies described immunostaining as an auxiliary diagnostic method. S100 was positive in 95.2% of cases, HMB-45 in 83.3% and Melan-A/MART-1 in 100%. According to previous studies, these markers can be also expressed in many diseases such as clear cell sarcomas, PEComas (angiomyolipomas, lymphangiomyomatosis, pulmonary ‘sugar’ tumors), renal cell carcinomas, and melanocytic schwannomas, among others (25,26). These studies highlight the importance of identifying the need for using more than one marker for diagnostic closure, since one marker alone is not enough to define melanoma malignancy.

In respect to treatment, most of cases underwent total parotidectomy, with a poorly described about recurrence (2 of 14 cases recurred). Wertz *et al*. (27) reported that total parotidectomy involves a reduced rate of recurrence of melanoma metastasis to the parotid gland and no increase in morbidity compared to total and partial parotidectomy. Interestingly, as approximately 70% of metastatic melanoma patients harbor mutations in oncogenes driving tumor progression (28), targeted therapy holds promise as an effective approach. Notably, the BRAF gene represents one of these oncogenic mutations observed in melanoma (29). Both animal studies and clinical trials have illustrated that BRAF monotherapy yields a high rate of objective response and enhances overall survival compared to chemotherapy in metastatic melanoma (7). None of the cases encompassed within this systematic review utilized these therapies. Nonetheless, there is an anticipation that these innovative treatments will witness a growing adoption.

Follow-up data were informed in 36 cases, and 41% of cases had death as the outcome. In fact, metastatic melanoma presented an obscure prognosis. A study of patients with metastatic melanoma reveled reported overall rates of 44%, 27%, and 17% in 1-, 2-, and 5-year, respectively (30). In our sample, 75% of patients were alive in 1 year, 55% in 2 years, and 38% in 3 years of follow-up. Whether the survival rates of metastatic cases within the major salivary glands are slightly better than the overall metastatic cases remain to be confirmed using larger samples and with longer follow-up periods. Male sex, nodular clinicopathological type, Breslow thickness exceeding 1mm, and the presence of ulceration was reported as a significant prognostic factors for metastasis in cutaneous melanoma in a multivariate analysis of 244 patients (31). According to the 8th American Joint Cancer Committee (AJCC) Staging Manual, the Breslow thickness stands as a pivotal determinant in assessing both the prognosis and staging of melanoma (32,33). This metric denotes the vertical depth of the tumor, gauged from the top of the granular layer of the epidermis to its deepest invasive extent, typically quantified in millimeters (33). Notably, Breslow thickness demonstrates a direct correlation with the propensity of melanoma to metastasize, with thicker lesions often indicative of a poorer prognosis. In the present study, factors such as sex, age, primary tumor site, and time to metastasis detection were explored, but found no significant associations.

Metastasis to salivary glands involves intricate mechanisms, including hematogenous and lymphatic routes, integrin and matrix metalloproteinase-mediated adhesion and invasion, and tumor microenvironment adaptations exploiting genetic mutations (34). Approximately 294 genes have been closely linked to melanoma metastasis (35), illustrating the complexity of understanding salivary gland metastasis (36). Comparative analysis is challenging due to genetic variability, immune responses, and unique tissue features, underscoring the need for comprehensive studies to deepen our understanding of these intricate oncological scenarios. These variables can profoundly affect metastatic behavior and offer potential avenues for targeted intervention. Nevertheless, the limited number of cases and often incomplete datasets underline the necessity for more comprehensive and detailed studies to enhance understanding and improve management strategies for these complex oncological scenarios.

The analysis of current survey data highlights limitations within the articles included in this systematic review. A noTable lack of data on recurrence and survival suggests the need for future studies to provide more comprehensive patient follow-up post-treatment. Although histopathological characteristics were extensively discussed, many articles merely labeled the condition as "metastatic melanoma" indicating a significant deficiency in histological characterization detail for metastatic melanoma in the major salivary gland. While articles lacking histopathological descriptions were not excluded due to the rarity of the condition, future reports should adhere to case and series reporting guidelines to enhance confidence in the published literature. This systematic review, primarily based on case reports, faces challenges including lack of standardization and heterogeneity in patient information. Consequently, the inherent heterogeneity in case reports precludes reliable meta-analyses of prevalence and/or association.

## Conclusions

Our findings confirm that metastatic melanoma of the major salivary glands is a rare condition, affecting more mild-age males. In histopathological terms, various cellular phenotypes have been described, although few reports have documented the presence of melanin pigment in the cells. Follow-up data indicated survival rates of 45% at 5 years. We emphasize that this is the first systematic review that allows us to learn more about the demographic and clinical characteristics specifically of metastatic melanoma of the major salivary glands.

- Other information

The systematic review followed Preferred Reporting Items for Systematic Reviews (PRISMA) guidelines (37) and had a registered protocol with National Institute for Health Research International Prospective Register of Systematic Reviews (PROSPERO) (number: CRD42022352499).

## Figures and Tables

**Table 1:** Summarize features of the cases of metastatic melanoma on major salivary glands.

Variable (Number of informed cases)		N (%)
Age, in years (47)	Mean±SD	56.3±21.5
	Range	may-85
Sex (47)	Male	33 (70.2)
	Female	14 (29.8)
Primary site of melanoma (46)	Skin in the head and neck region	29 (63)
	Orbital/periorbital region	11 (23.9)
	Skin in other part of body	6 (13.1)
First diagnosis (31)	Primary site	25 (80.6)
	Metastatic lesion	6 (19.4)
Time between first diagnosis and metastasis, in years (25)	Mean±SD	2.5±2.1
	Range	0.1-9.3
Metastasis site (47)	Parotid gland	44 (93.6)
	Submandibular gland	3 (6.4)
Pain (14)	No	8 (57.1)
	Yes	6 (42.9)
Evolution time, in months (14)	Mean±SD	4.6±3.8
	Range	0.5-12
Size, in centimeters (35)	Mean±SD	2.5±1.3
	Range	0.18-6
Treatment (38)	Total/radical surgery	25 (65.8)
	Superficial surgery	7 (18.4)
	Multiple	6 (15.8)
Follow-up, in months (36)	Mean±SD	22.8±28.4
	Range	1-148
Recurrence (14)	No	12 (85.7)
	Yes	2 (14.3)
Status (39)	Alive	23 (59)
	Death	16 (41)

SD, standard deviation.

**Table 2:** Histopathological and immunohistochemistry features of the informed cases.

Author, year of publication (Country)	Histopathological features	Immunohistochemical findings	
		Positive	Negative
Cochrane et al., 1993 (United Kingdom)	The cells possessed eosinophilic cytoplasm and oval nuclei with an open chromatin pattern and prominent nucleoli. There was both extracellular and intracellular accumulation of brown pigment	S-100	CK
Chhieng et al., 1999 (United States)	The tumor was highly variable in cellularity and composed of spindle cells arranged in fascicles and whorls. The neoplastic cells displayed mild to moderate atypia. Mitotic figures were frequent. Cytoplasmic pigmentation was absent. There was extensive intercellular collagenous deposition. A prominent lymphoid infiltrate, particularly in the perivascular areas, was noted. Perineural invasion was frequent	S-100	HMB-45
Prayson and Sebek, 2000 (United States)	All the 12 cases were composed of a generally diffuse arrangement of cells with abundant eosinophilic cytoplasm, large pleomorphic nuclei, and prominent nucleoli. In 8 tumors, the neoplasm was composed almost exclusively of cells with a more rounded appearance. Four tumors had a mixture of round and more spindle-shaped cells, with a predominance of rounded cells noted in 3 of these lesions. Melanin pigment was readily identifiable in 8 of 12 tumors. Areas of geographic necrosis were present in 7 tumors. Intravascular or intralymphatic involvement by tumor within the parotid gland was observed in 3 cases	S-100 (n=4), HMB-45 (n=1)	PanCK (n=3)
King et al., 2001 (United Kingdom)	The tumor consists of sheets of large pleomorphic undifferentiated cells typical for malignant melanoma. Pigment was seen in occasional cells	S-100	CK and LCA
Andreadis et al., 2006 (Greece)	Fascicles spindle-shaped cells with large pleomorphic vesicular nuclei, prominent nucleoli and clear-to-pale eosinophilic cytoplasm arranged in a storiform pattern. Multinucleate giant cells and lymphocytic infiltration were observed as well. The peri and intraparotid lymph nodes were infiltrated by malignant melanoma, whereas the adjacent parotid tissue was clear. Areas with melanin pigmentation, necrosis with hemorrhage and marked fibroblastic response were also seen	S-100, vimentin, HMB-45	-
Gottschaller et al., 2006 (Germany)	NI	S-100, HMB-45	-
Gross et al., 2008 (Israel)	The tumors were composed of diffuse arrangements of cells with abundant eosinophilic cytoplasm, large pleomorphic nuclei, and prominent nucleoli. Tumor cells varied from spindle-shaped cells to epithelioid cells	S-100 (5 cases), HMB-45 (3 cases)	HMB-45 (2 cases)
Elshenawy et al., 2011 (United States)	Most cells contained abundant granular cytoplasmic melanin pigment surrounding a single rounded nucleus. Binucleated cells were frequently seen in less than a quarter of the cell population, and multinucleation were rarely encountered. Nuclei contained easily visible pale prominent macronuclei. Focal nuclear irregularity was present; however obvious pleomorphism was not seen	MelanA/MART-1, HMB-45	LCA, CK and S-100
Masaoudi et al., 2013 (Canada)	NI	HMB-45	-
Neto et al., 2015 (Portugal)	Neoplasm composed of cells with abnormal nucleus/cytoplasm with round nuclei oval eccentric, with moderate pleomorphism and dense chromatin	S-100, vimentin, HMB-45	CK
Cengiz et al., 2016 (Turkey)	Epithelioid neoplastic cells with foamy cytoplasm, marked cytologic atypia, nuclear grooves, large eosinophilic nucleoli, and abundant atypical mitotic figures. Also, tumor necrosis was observed in the focal area	S-100, vimentin, MelanA/MART-1, HMB-45	-
Kılıçkaya et al., 2016 (Turkey)	NI	HMB-45, S-100, MelanA/MART-1	PanCK
Agrawall et al., 2017 (India)	NI	HMB-45	-
Kumar et al., 2018 (India)	High-grade malignant tumor with spindle and epithelioid cytomorphology and prominent nucleoli	S-100, MelanA/MART-1, HMB-45, SOX-10	PanCK
Sensu et al., 2020 (Turkey)	Pleomorphic cells with irregular nuclear contours, coarse chromatin and prominent nucleolus were forming discohesive clusters	MelanA, HMB-45, S-100	PanCK, CD45, SMA
Ahadi et al., 2021 (Iran)	Malignant neoplasm composed of fascicles of atypical spindle-shaped cells with large pleomorphic vesicular nuclei, prominent nucleoli, and clear-to-pale eosinophilic cytoplasm arranged in a storiform pattern. Multinucleated giant cells and lymphocytic infiltration were observed as well. Notably, mitotic activity was about 4/10 high-power field	HMB-45, S-100	MyoD1, Desmin, PanCK, CK5/6
Naseem et al., 2022 (Pakistan)	Clusters of large clear cells resembling a typical melanocytes. These pigmented nests of atypical melanocytes along with melanophages were seen invading the superficial dermis and were also involving upper part of epidermis in the form of pagetoid spread. The tumor-stroma interface showed a marked inflammatory response and fibrosis	HMB45	-

NI, not informed; CK, cytokeratin; HMB, Human Melanoma Black; LCA, Leukocyte Common Antigen; MART, melanoma antigen recognized by T cells.

**Table 3:** Summarize immunostaining features of the cases of metastatic melanoma on major salivary glands.

	S-100 (21)	HMB-45 (18)	Melan-A/MART-1 (5)	PanCK/CK (9)	Vim (3)	LCA (3)	SOX10 (1)	SMA (1)	CD45 (1)	Desmin (1)	MyoD1 (1)
Positive	20	15	5	0	3	0	1	0	0	0	0
Negative	1	3	0	12	0	3	0	1	1	1	1

HMB, human melanoma black; MART-1, Melanoma-associated antigen recognized by T cells-1; Vim, vimentina; LCA, leukocyte common antigen; SOX10, Sry-related HMg-Box gene 10; SMA, smooth muscle actine; PanCK, pancytokeratin; MyoD1, Myogenic Differentiation 1.
